# Arbovirology and Cold War Collaborations: A Transnational History of the Tick-borne Encephalitis Vaccine, 1930-1980

**DOI:** 10.1093/jhmas/jrad054

**Published:** 2023-09-08

**Authors:** Anna Mazanik

**Affiliations:** Medical University of Vienna, Vienna, Austria

**Keywords:** Tick-borne encephalitis, Virology, Vaccines, Zoonosis, Cold War science, University of Vienna, MRE Porton Down

## Abstract

This article analyzes the history of immunization against tick-borne encephalitis (TBE) and specifically the processes that led to the creation and application of TBE vaccines in the Soviet Union and Austria. Rather than presenting the development of TBE vaccines from the perspective of national scientific schools, the article investigates their history as a transnational project, focusing on the connections among the Soviet Union, Czechoslovakia, Austria, the United States, and the United Kingdom. It argues that biomedical research on TBE was profoundly intertwined with political and military agendas and depended on civil international cooperation as well as Soviet, American, and British military concerns, infrastructures and funding. The article consists of four parts that discuss (1) the identification of the TBE virus and the creation of the first TBE vaccine in the Soviet Union in the 1930s; (2) the internationalization of TBE research and vaccine development in the 1940s-1960s; (3) the history of TBE research and virology in Austria in the 1930s-1960s and the role of the US military funding; and (4) the cooperation of Austrian virologist Christian Kunz with the Microbiological Research Establishment Porton Down in the UK leading to the development of the Austrian/British vaccine against TBE in the 1970s.

Tick-borne encephalitis (TBE) is a viral disease of the central nervous system that can lead to severe, disabling, or even deadly symptoms, including paralysis, sensory impairments, or respiratory failure. The disease is transmitted by *Ixodes* ticks, endemic in non-tropical Eurasian forests and, after Lyme disease, it is the second most prevalent tick-borne disease in Europe. The disease develops as a result of a bite from a tick carrying TBE virus (TBEV) or, in rare cases, of consuming unpasteurized dairy products from TBEV-infected animals.^1^ Approximately 10,000-12,000 cases are reported annually but the World Health Organization (WHO) believes those numbers to be underestimated.^2^

In the absence of a specific therapy, vaccination is considered to be the best measure against TBE. It is advocated by expert groups, national regulators, and international organizations. There are currently five vaccines against TBE, produced in Austria, China, Germany, and two in Russia. The first TBE vaccine was developed in the Soviet Union in the 1930s but today international recommendations usually reference the Austrian experience.^3^ In 1981, Austria started a nationwide vaccination campaign against TBE that continues until today. By 2001, more than 85% of the Austrian population had been vaccinated.^4^ No other country in the world has shown comparable mobilization.

This article focuses on the first half a century in the history of TBE immunization (1930-1980) and specifically on the processes that led to the development and application of TBE vaccines in the Soviet Union and Austria. In the medical and public narrative in both Russia and Austria, the TBE research and vaccine development are celebrated as a major achievement in the twentieth-century history of the country’s medical science. Both narratives are based on the claim of being the first: in Russia of being the first to isolate the TBE virus, to identify tick as a vector, and to develop the first vaccine; in Austria of being the first to describe the disease in its European manifestation and to invent the first vaccine suitable for mass immunization in Europe.^5^ Both cases are the stories of scientific success for public good framed in Promethean and national terms, with no relation to each other or to transnational processes.

Studies in the history of science, however, have repeatedly demonstrated that the reality of science and medicine is much more complex than such narratives suggest. Rather than presenting the development of TBE vaccines from the perspective of national scientific schools and civil public health programs, this article investigates their history as a transnational project, focusing especially on the connections among the Soviet Union, Czechoslovakia, Austria, the United States, and the United Kingdom.

A number of historical studies have shown that military concerns and Cold War geopolitics had a huge impact on the trajectory, scope, and content of science in many domains, including biomedicine, and highlighted the importance of US funding for the development of science in Western Europe.^6^ However, existing scholarship still weighs heavily towards the American, Western European and, to some extent, Soviet experience.^7^ Scholarly interest in Central European biomedicine and Cold War scientific exchange in Central Europe is only beginning to emerge and little is known of how defense concerns influenced biomedical sciences in the region.^8^

Building on this scholarship, the article reveals that the study of TBE was tightly entangled with political and military agendas of the (long) Second World War and the Cold War. It argues that TBE research developed at the intersection of scientific internationalism and Soviet, American, and British military interests. The article is divided into four parts that together expose the complex global political and scientific processes and cross-border collaboration behind the development of the TBE vaccines. The first part focuses on the identification of the TBE virus and the creation of the first TBE vaccine in the Soviet Union in the 1930s with the background of a military conflict with Japan. The second part discusses the internationalization of TBE research in the 1940s-1960s and the development of the new tissue-culture vaccine. The third part studies the history of TBE research in Austria in the 1930s-1960s, revealing its transnational connections and especially the importance of US military funding for Austrian arbovirology. Finally, the last part is dedicated to the cooperation between Austrian virologist Christian Kunz and the Microbiological Research Establishment Porton Down in the UK and the development of the Austrian vaccine against TBE in the 1970s, which as the article shows, should rather be called the Austro-British.

## The Soviet Vaccine, 1930s-1940s

In the 1930s, an outbreak of a severe paralytic disease was recorded in the Soviet Far East, the region that was gaining strategic importance with the background of Soviet military tensions and border conflicts with Japan, following the Japanese occupation of Manchuria in 1931-1932. In 1937, at the request of the Military-Sanitary Department of the Red Army, the Soviet *Narkomzdrav* (People’s Commissariat of Health) organized a scientific mission, led by a Jewish virologist Lev Zilber, to investigate reports of the unknown disease in the region of Khabarovsk. Zilber’s expedition established the viral etiology of disease, which soon became known in Russian as tick-borne encephalitis (*kleshchevoi entsefalit*) and in English as Russian spring-summer encephalitis; it isolated the causative virus and identified the *Ixodes* tick as its vector. That discovery coincided with the peak of Stalinist purges, and upon their return to Moscow Lev Zilber and two of his female colleagues, virologist Alexandra Sheboldaieva and epidemiologist Tamara Safonova, were arrested and sent to the Gulag on the accusation of being Japanese spies, as a part of the broader repressive campaigns against biologists and medical researchers both civilian and military. The names of Zilber and Sheboldaieva were excluded from the list of the co-authors of the expedition publications and would only be re-introduced after Stalin’s death.^9^

In 1938-1940, the expeditions to study TBE continued under the new leadership of parasitologist Evgeny Pavlovsky, virologist Anatoly Smorodintsev, and epidemiologist Isaak Rogozin. The isolated TBE virus became the key to studies of the environmental circulation of arthropod-borne viruses, later developed by Pavlovsky in his natural nidality theory, which applied the ecological niche approach to the study of zoonoses. Pavlovsky’s theory not only became a dominant framework in Soviet public health at least until the 1980s but also provided the pattern for initial research on related vector-borne viruses across the globe and is sometimes considered to be one of the theoretical precursors of the science of landscape epidemiology and One Health agenda.^10^

The subsequent studies of the 1940s have changed the understanding of TBE localization as the disease was revealed in the Urals and the European part of the Soviet Union. In 1942-1943, TBE was reported among the soldiers of the Red Army’s Volkhov Front between Leningrad/St. Petersburg and Kalinin/Tver’, close to the country’s northwestern borders. However, severe symptoms seemed to appear much more rarely in western regions.^11^ According to today’s knowledge, tick-borne encephalitis is caused by three main subtypes of the TBE virus, with the European subtype being the mildest, with a case-fatality rate below 2%, and the Far-Eastern subtype associated with the most severe patient outcomes and the highest fatality.^12^ Although in the last decades the Far-Eastern subtype has been in decline,^13^ it was exactly the variant that Soviet scientists first encountered in the 1930s and that determined the early perceptions of TBE as extremely dangerous.

The attempts to develop preventive measures began soon after the discovery of the virus. Although the ultimate goal was to have prophylaxis for the military and the general population, the urgent need to protect the laboratory researchers working with the virus provided an important motivation. In 1937-1939 three scientists died of TBE, and at least four more contracted the virus and had a severe course of the illness, with symptoms such as blindness, deafness, and paralysis.^14^

Despite the military importance of TBE, the participation of the military physicians in the field expeditions, and the existence in the Stalin-era Soviet Union of a powerful military-biological complex that managed to produce several novel vaccine preparations,^15^ the development of the TBE vaccine was a civil project. The remarkable aspect of the early TBE vaccine development was that it was primarily done by women, revealing the significant feminization of Soviet microbiology already in the 1930s.^16^ The work on the vaccine started in 1938 and was led by two female virologists, both affiliated with the Moscow’s All-Union Institute of Experimental Medicine: Nadezhda Kagan in Moscow and Elizaveta Levkovich, who was a deputy head of Zilber’s expedition, in the field in the Khabarovsk region. The laboratory where the research was conducted was also staffed with female personnel. In the autumn of 1938 Kagan contracted TBE and died, and Levkovich took over her work. Two months later, a young laboratory technician Natalia Utkina died after contracting TBE and the following year her (female) cousin working in the same lab was exposed to the virus but eventually recovered after several months in the hospital. Female bodies were also the first to try the new vaccine when Levkovich and her assistant Galina Zorina-Nikolaieva tested the vaccine on each other in 1939.^17^

The first TBE vaccine was based on the Sofyin strain of the Far-Eastern subtype of the virus and was prepared from the brain suspension of infected mice that was inactivated with formalin. To check the efficiency of the vaccine, larger trials were conducted in 1939 on the population of the endemic area around the village of Obor in the Khabarovsk region. At that time the local population consisted largely of deported “special settlers” and Gulag forced laborers and had high exposure to infected ticks because of the work in wood logging. The accounts on the exact number of participants and the course of the trials differ. The 1941 publication of the trial results reports 925 vaccinated and a control group of 1185 unvaccinated persons who were distributed across four locations and had a comparable age, gender, and occupational composition. This account does not mention the legal status of the participants but claims that both groups were offered “sanitary explanation” about the trials although it is unclear what exactly that involved. Another account, the 2001 memoir of the expedition member Alexei Shapoval, published 60 years after the described events, speaks of 1987 vaccinated and explicitly states that they were inmates of the NKVD forced labor camp while another camp with 2387 prisoners in the same area was used as a control group. Such composition of participants would suggest that the involvement in the trial was not voluntary. Luckily for those vaccinated, both accounts agree that the vaccine seemed to be successful and offered some protection against the disease. The official publication reported only 2 mild TBE cases among the vaccinated compared to 27 cases and 7 deaths among the control group; Shapoval recalled 9 mild TBE cases among the vaccinated compared to 37 TBE cases and 12 deaths in the control group.^18^ Those limited trials were used as a confirmation of the new vaccine’s efficiency and it was allowed as a prophylactic measure for exposed scientists as well as the population of the endemic areas of the Khabarovsk region.^19^

The case-fatality rate of TBE observed in the early trials (27-32% in the control group) was dramatic but it is unclear to which extent it can be attributed to the virus alone. Medical research in the Gulag has recently come to the attention of historians, who revealed the “conspicuous silence” of Soviet scientists, many of whom were also prisoners, about the social context of their research subjects, when any references to camps, starvation, or ruthless exploitation were avoided.^20^ In the case of TBE research, the concealment of the camp context had not only ethical but also empirical implications. The health status and post-infection survival chances of forced laborers or settlers had been severely compromised by very poor nutrition, vitamin deficiencies, exhaustive work, lack of healthcare, as well as abuse, torture, and other traumatic experiences connected to arrest, deportation, and imprisonment. However, Soviet scientists did not reflect on how those factors could have influenced the striking TBE mortality and morbidity they observed and attributed them exclusively to the properties of the virus, reinforcing the image of tick-borne encephalitis as highly lethal.

## Internationalization of the TBE Research and the New TBE Vaccine, 1940s-1960s

The 1940s and 1950s witnessed an explosion of interest in tick-borne encephalitis. One reason was the dissemination of Soviet studies and the increased international attention to Soviet biomedicine more generally. WWII reversed the isolationist tendencies of Soviet science, evident in the late 1930s, and re-opened opportunities for exchange between Soviet and Western scholars, especially in natural sciences.^21^ Virology became one of the fields of such cooperation.

As the findings of Soviet TBE research were quickly published in widely available academic outlets and not only in Russian but also in German and English, it allowed for international recognition of Soviet virology and stimulated interest in tick-borne viruses.^22^ Already in 1941, Soviet scientists sent a strain of TBE virus to the Rocky Mountain Laboratory of the US Public Health Service, providing the material that was used for the early TBE experimentation in the US.^23^ In 1944, Soviet virologists Anatoly Smorodintsev and Valentin Soloviev, both of whom then specialized in TBE research, visited the US as guests of the US Typhus Commission and the Rockefeller Foundation, reciprocating the visit of the American medical delegation to the Soviet Union a couple of months earlier. During the visit, they met several American virologists, including Albert Sabin and Joseph Smadel, the Director of the Department of Virus and Rickettsial Diseases at the Walter Reed Army Institute of Research, and maintained professional relations with them, including the exchange of publications and viral samples in the fields of mutual interest, such as TBE and mosquito-borne Japanese encephalitis (both diseases were seen as a health hazard in the Pacific region and therefore of particular interest to the US and the Soviet Union in their military operations against Japan).^24^

The other reason for the international interest in tick-borne encephalitis was the apparent emergence of this disease in Central and Eastern Europe during and immediately after the war. The early accounts initially assumed that encephalitis was brought by the moving armies.^25^ It is possible, however, that the increased visibility of this disease was connected to the social and environmental changes caused by the war. The disruption of agriculture likely led to the proliferation of tick hosts in abandoned or unmanaged fields and meadows. The displacement, malnutrition, and decreased health status of the population in the extreme circumstances of the war could have also contributed to the frequency of symptomatic infections and severity of the disease course.^26^

In 1949, scientists at the Czechoslovak National Institute of Health Frantisek Gallia and Josef Rampas, together with Ludvik Hollender from the Bulavka City Hospital, isolated the first strains of tick-borne encephalitis in Central Europe that corresponded to the low-mortality western variant of the TBE virus from the Soviet Union.^27^ This variant of the virus became known as the Czech and then as the Central European encephalitis virus. Throughout the 1950s, scientists in Czechoslovakia, Hungary, Poland, Austria, Bulgaria, Yugoslavia, the Netherlands, Finland, and Sweden isolated the virus in their regions and identified it as a cause of already familiar diseases of previously unknown etiology, such as “meningitis serosa epidemica” in Austria and “Kumlinge disease” in Finland.^28^

The fact that many of these scientists were based in Eastern bloc countries, were able to understand Russian and to follow scientific literature from the Soviet Union, where tick-borne viruses and viral encephalitis became a major focus of virology, helped foster regional scientific cooperation and validate TBE research as a relevant - and ideologically safe - field of inquiry in a tense political climate of the socialist transformation and the early Cold War. Czechoslovakia emerged as a key center of research on Central European encephalitis, with Czechoslovak scientists doing pioneering studies on epidemiological, biochemical and biophysical aspects of the virus, as well as its local hosts and reservoirs, its transmission by migratory birds and through unpasteurized goat’s milk. This research was concentrated at the new Institute of Virology of the Czechoslovak Academy of Sciences in Bratislava, founded in 1952 and led by Slovak virologist (and 1946-1948 former Rockefeller Foundation fellow) Dionyz Blaskovic.^29^ The Institute managed to profit from the substantial state support for the development of applied natural sciences in Czechoslovakia as well as limited internationalism and opportunities for cooperation with western scientists that opened up with de-Stalinization.^30^

Importantly, TBE research was not just an Eastern bloc project but also an area of intensive scientific exchange across the Iron Curtain. The identification in the 1940s and 1950s of other tick-borne viruses dangerous to humans and related to the Russian spring-summer encephalitis virus (for example, Langat virus in Malaysia, Kyasanur Forest Disease virus in India, Powassan virus in Canada) revealed that the problem was global in scale. As the natural habitats of TBE and other tick-borne viruses and their hosts crossed the political boundaries of the increasingly polarized world, their study provided a forum for cooperation in the still small but booming field of arbovirus research. When in 1960 the first international symposium on the viruses of the tick-borne encephalitis complex was held in Smolenice, Czechoslovakia, about 60 km from the border separating the Eastern bloc from Austria, it brought together 76 scientists from 12 countries in Europe (both socialist and capitalist), Asia, and North America.^31^

With the rise of the international health movement, the WHO emerged as a particularly important arena for collaborative investigations across the Cold War borders and by the early 1960s allowed the transition from bilateral exchange programs to multilateral scientific cooperation. Using the opportunity of the International Congress on Tropical Medicine in Lisbon in 1958, the WHO organized an informal meeting of arbovirus experts on the systematic exchange of scientific information. As a direct result of that initiative, the WHO held a series of meetings concerning international policy on arbovirus research (Scientific Group on Virus Research in November 1958; Scientific Group on Immunological and Haematological Surveys in December 1958; Scientific Group on Research on Birds as Potential Disseminators of Arthropod-borne Viruses in March 1959) and eventually set up a Study Group on Arthropod-borne Viruses in September 1960. Although in its original composition the group was dominated by Western scholars, it also included Dionyz Blaskovic from the Institute of Virology in Bratislava as a Vice-Chairman and Antonina Shubladze from the Ivanovski Institute of Virology in Moscow, the only female member of the group (and also a former member of Zilber’s 1937 expedition to study TBE). By the mid-1960s the WHO Arbovirus Study Program established a global network of arbovirus reference centers, while the reports of the group provided a kind of an international guide for arbovirus research.^32^ One of the outcomes of this international cooperation was a standard nomenclature and a more nuanced classification of the viruses based on their antigenic similarity. In 1963, the WHO endorsed the abbreviation “arbovirus” to signify the “arthropod-borne virus,” finalizing the establishment of the field of arbovirology.^33^ In 1964 American virologist Delphine Clarke from the Rockefeller Foundation Virus Laboratories suggested that Central European and Russian spring-summer encephalitis virus should be considered variant subtypes of the same tick-borne encephalitis virus, distinct from other viruses of the complex (Langat virus, Powassan virus, Kyasanur Forest Disease virus, Omsk Hemorrhagic Fever virus), and that was the view that has prevailed ever since.^34^

Throughout the early post-war decades the Soviet mouse-brain formalin-inactivated vaccine remained the best available protection against TBE on both sides of the Iron Curtain. The Walter Reed Army Institute of Research stimulated the production of the mouse-brain vaccine that was used to immunize laboratory personnel working with arboviruses. In 1957, during the outbreak of the Kyasanur Forest disease in India (which at that stage was believed to be closely related to Russian tick-borne encephalitis), the Walter Reed Institute sent 50,000 doses of this vaccine to protect the population of affected areas.^35^

Meanwhile, the widespread application of the vaccine in the Soviet Union revealed frequent negative reactions towards the mouse brain components, and the Soviet scientists started experimenting with alternative vaccine technologies, taking advantage of the new international collaborations that opened up following Stalin’s death. This research was concentrated at the new Institute of Poliomyelitis and Viral Encephalitis in Moscow, founded in 1955 and headed by Mikhail Chumakov, who too had been a member of Zilber’s 1937 expedition where he survived but was left disabled by a severe TBE infection.^36^ The Institute’s team also included Elizaveta Levkovich and other scientists involved in the TBE research from the 1930s.

The Institute of Poliomyelitis and Viral Encephalitis quickly emerged as perhaps the most internationally visible -- and internationally networked — organization in Soviet biomedicine. The Soviet-American scientific cooperation, which allowed Soviet virologists to travel to the US in the 1940s, was interrupted by the onset of the Cold War but resumed in 1956 when a group of Soviet virologists, led by Mikhail Chumakov, his wife Marina Voroshilova, and Anatolii Smorodintsev visited the US to study the new American vaccines against polio. The most famous outcome of ensuing cooperation was the large-scale trials of Sabin’s oral polio vaccine in the Soviet Union that validated its safety for mass immunization around the world and contributed to building the international reputation of Mikhail Chumakov. That scientific exchange also covered the field of tick-borne viruses that were one of Chumakov’s key interests.^37^ Chumakov’s reputation in arbovirus research was connected not only to TBE but also to his work on other tick-borne viral diseases such as Omsk hemorrhagic fever (whose agent he isolated in 1947) and Crimean-Congo hemorrhagic fever. He also joined the WHO Arbovirus Group and, together with his team, contributed to writing the WHO guidelines.^38^

In 1959-1960, the Institute of Poliomyelitis and Viral Encephalitis began the production of a new vaccine against TBE based on the cell culture of chicken embryos to avoid the severe reactions to the mouse-brain components of the previous version of the vaccine. The new vaccine was also based on the Sofyin strain of the Far Easter subtype of the TBE virus that was inactivated with formaldehyde and purified by separation, followed by clarification and sterile filtration. Throughout the 1960s, the Institute carried out large-scale human trials of the vaccine, which also involved cooperation with the Steklov Institute of Mathematics of the USSR Academy of Sciences to take advantage of the new possibilities in data analysis. The immunogenicity of the vaccine was first tested on 42 volunteers without previous exposure to TBE by the standard neutralization test in mice and the hemagglutination inhibition test. Afterwards its effectiveness was checked in the field when its ability to prevent the infection in the vaccinated group was compared to the old mouse-brain vaccine and the unvaccinated control group. In 1961, 26,000 persons received the new vaccine, 52,000 the old vaccine, and the unvaccinated control group comprised 115,000 persons. 95,500 persons from all three groups submitted epidemiological questionnaires about their behavior, visits to forests, travel history, and frequency of tick bites, and this data later went through computer analysis to calculate the exposure risks. The three groups were monitored for all symptomatic forms of infection, regardless of whether or not they led to hospitalization. These controlled trials lasted for four years to check for the length of the vaccine-induced immunity and the impact of re-vaccination and the annual fluctuations in tick activity, and the observations of the vaccinated group continued also afterwards. The vaccination status was blinded to epidemiologists working and the field, and the vaccination record was provided to a special committee that met at the end of the epidemic season.^39^

In contrast to the late 1930s, the development of the new TBE vaccine was a project open to and profiting from international collaboration. Not only was the leadership of the Institute well integrated into the international scientific networks, but also the trials themselves involved foreign scientists. Chumakov’s team included Czechoslovak virologist Helena Libikova and five of her co-workers from the Institute of Virology in Bratislava and American virologist Jakob Brody from the US National Institute of Health, who spent six months in the Soviet Union in 1962 as an exchange scientist at the Institute of Poliomyelitis and Viral Encephalitis in Moscow to work on TBE. Brody’s reports mention that during his stay the Institute had between 10 and 20 foreign workers and that Soviet scientists from the Institute also went abroad, including to the US and the UK.^40^

This vaccine was much better tolerated than the mouse-brain vaccine and was also more effective. However, the field trials showed that its immunogenicity in real life was lower than it was assumed on the basis of the initial tests with volunteers. Furthermore, it required a very complex and long schedule of four-fold vaccination and three boosters and showed frequent breakthrough infections if the course was not followed. As a result, mass immunization demanded outstanding organizational capacities and had limited effectiveness in real-life situation, especially at the beginning. In the Soviet Union, although the vaccine continued to be used in regional immunization campaigns, it did not become the favored defense against the disease. The 1950s and 1960s was a time of intensive colonization and industrial development of Siberia and the Soviet Far East when hundreds of thousands of immunologically naive migrants relocated to the regions endemic of dangerous TBE variants. In this situation, the preference was given to the environmental forms of protection targeting not the exposed persons but the vector, when the areas around the settlements were regularly sprayed with chemical acaricides, particularly DDT. This method remained widely used throughout the country until the end of the Soviet period, even after the new generation of the Soviet TBE vaccine was licensed in 1982.^41^

The Soviet vaccine experiments and the development of the new tissue-culture vaccine attracted international attention, and not only among the narrow groups of scientists but also public authorities. In the early 1960s, the Austrian Social Ministry (BMfsV) sent a delegation to the Soviet Union to explore the possibilities of wider immunization against TBE. The delegation studied the mouse-brain and the tissue culture vaccines but both were eventually deemed unsuitable for mass vaccination in Austria due to their reactogenicity (mouse-brain vaccine) or insufficient effectiveness (tissue-culture vaccine) and the rarity of severe symptoms caused the European TBE variant compared to the variants common in the Soviet Union.^42^

In the 1960s in Central Europe, similar to the Soviet Union, TBE vaccination was not seen as the main protective mechanism against this disease. In his 1967 overview of TBE and public health for the WHO Bulletin, Dionyz Blaskovic did not recommend mass vaccination for the general population in Central Europe but only for the specific highly exposed groups. Instead, he proposed focusing on environmental methods that reduced the number of ticks and interrupted the circulation of the virus in nature, such as the management of meadows, shrubs, and footpaths and the limited use of DDT, as well as awareness-raising and individual protection with clothes and repellants.^43^ It was not until the appearance of the new vaccine in Austria that the immunization would become the preferred instrument against TBE in the region.

## Austrian TBE Research and Cold War Politics

In 1931, Austrian physician Hans Schneider from the hospital of Wiener Neustadt near Vienna reported the epidemic of “acute serous meningitis” in Lower Austria and noted the seasonality of this disease which peaked in warm summer months.^44^ Schneider’s study is considered to be the first clinical description of TBE. The ISW-TBE, the organization co-founded and chaired by Austrian scientists and usually holding its meetings in Vienna, counts Schneider’s publication as the beginning of TBE as a clinical entity.^45^ As it often happens with clinical descriptions that predate the discovery of the disease agent, this beginning has been disputed because some clinical symptoms that occur in TBE caused by the eastern viral subtypes had been described earlier in Russia.^46^

However, for more than two decades the etiology of Schneider’s disease remained unclear and its explanation was a result of a transnational — rather than local Austrian — effort. Throughout the 1940s and early 1950s similar epidemics of meningoencephalitis were recorded in other parts of Austria and elsewhere in the region, but because the agent was not identified and because the symptoms of TBE can be confused with other neurological viral infections, especially polio, the attribution of clinical cases and entire outbreaks to this disease was very problematic. Until the mid-1950s Austrian medical scientists discussed various etiologies, transmission routes, and predisposing factors for Schneider’s meningoencephalitis, including mosquito-borne and person-to-person transmission.^47^ The isolation of the TBE virus in Czechoslovakia in 1949, discussed above, offered a key to understanding encephalitis cases in various countries of Central Europe but it took several years until that finding was used to explain the outbreaks in Austria.

It was not the Austrian but the Dutch scientists who first linked Austrian meningoencephalitis to the viruses of the TBE complex. In 1954 a team of microbiologists from the University of Leyden, led by Jacobus Dirk Verlinde, isolated the virus from the brain samples of five fatal cases from the meningoencephalitis epidemic in south-eastern Austria from summer 1953, which they had received from the Neuropsychiatric Clinic in Graz. They showed its similarity to the strains of the Russian and Czech tick-borne encephalitis virus, obtained respectively through the institutions in France and the UK, as well as the meningoencephalitis virus isolated in Yugoslavia (from the area near the Austrian border) in 1953 and obtained from Zagreb.^48^

In 1957 this finding was further confirmed by Hans Moritsch from the Hygiene Institute at the University of Vienna and J. Krausler from the Neunkirchen Hospital, who retrospectively connected Schneider’s disease that had been clinically and epidemiologically observed in Lower Austria for more than 20 years to the viruses of the TBE complex. Moritsch was, however, skeptical about the tick bite as the key mode of transmission (as well as about other findings that were based primarily on Soviet studies) and did not exclude the transmission by mosquitoes. Moritsch suggested to rid the name of the disease from any geographical reference and instead focus on seasonality, calling it *Frühsommer-Meningo-Encephalo-Myelitis* or *Frühsommer-Meningoencephalitis* (FSME, literally “early summer meningoencephalitis”), which remains the standard name of this disease in German language until today.^49^ In the years following this publication, TBE research occupied a central place in Moritsch’s mostly locally-oriented scientific work, allowing him to emerge as Austria’s main expert in arboviruses and an important adaptor of the global scholarship for the German-speaking audience.^50^

At that stage virology was still relatively weak in Austria, falling behind not only Western scientific schools but also those on the other side of the Iron Curtain. In postwar decades the Austrian scientific establishment, reeling from the damage caused by the loss of Jewish professionals, the war, and the occupation, experienced a certain provincialization and had to undergo significant personnel and organizational changes to adjust to new political realities. For a long time, the material base of Austrian biomedicine remained very poor, lacking the most basic supplies, and fundamental science was almost put to a halt.^51^ Unlike Bratislava or Moscow, Vienna did not have its own separate center of virology but instead only a small unit within the University of Vienna’s Institute of Hygiene, which from 1962 was chaired by Hans Moritsch. According to the memoirs of his friend, Slovak-American virologist Jan Vilcek, Moritsch felt unsatisfied by scientific facilities in Vienna and depended on and benefitted professionally from close relations to Dionyz Blaskovic from the Institute of Virology in the neighboring Bratislava (Bratislava and Vienna are situated only 60 km apart). In the early 1960s Blaskovic was a very influential scientist both nationally and internationally; he was not only the leading figure of TBE research in Central Europe, but also the head of the Slovak Academy of Sciences, the vice-chairman of the WHO Arbovirus Group, the General Secretary of the International Council of Scientific Unions, and the member of the International Biological Program.^52^ When in 1964 Vilcek and his wife unexpectedly used a private invitation from Moritsch to visit him in Vienna as an opportunity to defect to the West, Moritsch was apparently worried that it would damage him professionally by putting an end to his close relations to Blaskovic and other virologists in Czechoslovakia.^53^

However, there were other factors that could have compromised Moritsch’s reliability in the eyes of the Czechoslovak authorities and that could have added to his concerns, as his professional ties extended both to socialist and capitalist blocs and served not only civil scientific cooperation but also military interests. In 1960 Moritsch was contracted by the US Army Research and Development Program to carry out a study of tick-borne encephalitis, marking the beginning of the at least 13-year cooperation between the US military and Austrian virologists in the field of TBE research.^54^

The interest of the US Army in tick-borne encephalitis was evident already since WWII and its military operations in the Far East but the onset of the Cold War gave that interest a new edge. As the Soviet Union turned from an ally into an archenemy, its research in microbiology and, in particular, on zoonotic infectious agents became an object of continuous military monitoring and fears of potential weaponization. The development of the Soviet biological weapons program throughout the 1950s and 1960s is still poorly known because the archival material remains classified. The existing scholarship, based on the scarce available accounts, suggests that during the Khrushchev period the program largely maintained a defensive character, that weaponization experiments with encephalitis virus started only in the late 1960s in response to the weaponization of the Venezuelan equine encephalitis virus in the US, and that the interest in the military use of the arthropod-borne viruses soon waned because of the difficulty of mastering both the pathogen and the vector population in the field situation.^55^ However, already in the early 1960s US intelligence considered tick-borne encephalitis virus a possible bioweapon threat, even though the direct evidence of such a program was lacking. For example, the 1964 CIA report on Soviet capabilities and intentions with respect to biological warfare named TBE virus one of eight likely candidates of weaponization in the Soviet Union because of extensive Soviet research on the virus and its endemicity in the country, which might give Soviet troops a possible immunological advantage.^56^

At the same time, even without Soviet weaponization, TBE was a possible military threat as the US Army was also concerned with naturally occurring infections that posed risks to its personnel. The emergence of Central Europe as a borderland between the two military blocs and the identification of multiple TBE endemic foci across the region turned the disease into a potential health hazard for the US troops that were already stationed or would have to be deployed there should the conflict move to an open military confrontation. Considering that TBE was not endemic in the US and that most of the research on TBE in Central Europe was concentrated in the socialist countries and was likely serving Soviet interests, supporting further TBE investigations in Austria appeared worthy of US military sponsorship.

Historians have revealed the huge role that American funding played in the reconstruction and development of Western European science and its instrumentalization for building American hegemony. John Krige has argued that American organizations did more than just share science and promote the values of liberal capitalism; instead, they attempted and often succeeded in reconfiguring European science in many domains.^57^ Although Austria officially remained a neutral country, it appears that Austrian virology was a part of this process.

Between 1960 and 1972, scientists from the University of Vienna carried out biological, immunological, biophysical, biochemical, epidemiological, and ecological studies of the TBE virus and other arboviruses for the US Army.^58^ Although this cooperation allowed Moritsch to get funding for his scientific ambitions — the goals of the original 1960 contract looked remarkably similar to the research program that Moritsch outlined in his 1957 publication — it probably also cost him his life. In 1965, the 41-year old Moritsch died of encephalitis.^59^

The US Army Research and Development Program financed hundreds of scientific projects of direct or not so direct military interest, many of them abroad. The purpose of foreign grants was to perform studies in the fields that were not available in the US, to receive access to the European scientific and technical community, obtaining information that was difficult to get otherwise. Yet, TBE research in Austria apparently was not just any project. When in 1966 General Austin Betts, Chief of US Army Research and Development, had to explain military spending, including foreign contracts, in front of the US Congress, he chose Austrian TBE research as one of six examples to justify the funding of the entire foreign program. This suggests that the program leadership viewed Austrian contracts as successful and useful to the US — or at least as having the potential to appear as such. Moritsch’s tragic death presented a handy argument to confirm that the virus was extremely dangerous and that research on it should be outsourced to foreign scientists.^60^

In Austria, the impact of the cooperation was even more perceivable. When, shortly before his death, Moritsch wrote an overview of the historical and contemporary status of virology in Austria, most of the text was devoted to TBE and about one-third to the studies performed under US contracts (although that cooperation was not mentioned), suggesting the importance of that funding for the entire discipline.^61^ Furthermore, continuous American funding allowed TBE to remain the core interest of virology in Vienna even after Moritsch’s death when the program was taken over by his younger colleague Christian Kunz, then in charge of the virology unit within the Hygiene Institute.

Kunz represented a new generation of Austrian scientists that was much better integrated into the international scientific networks. He was trained in Germany and, as a Rockefeller Foundation fellow in 1961-1962, in the US where he also met his American wife.^62^ The contracts with the US Army Research and Development Office do not specify the exact amount of funds allocated, but it is evident that under Kunz the research program became much more complex and larger in scope, although it is unclear whether the drive behind this growth was the increase of available funding, specific American interests or Kunz’s own ambition. In addition to the epidemiological and clinical studies of TBE in Austria, started already by Moritsch, in the late 1960s the work under US contracts expanded to include other viruses (Tahyna virus, Marburg virus, Tribec virus), their prevalence among the human and animal population not only in various parts of Austria but also in West Germany, Switzerland, Slovakia, Turkey and Cameroon, their potential vectors, reservoirs and transmission routes, purification, concentration, and attenuation techniques, possible treatments, and prevention strategies. That research was beneficial to Kunz’s career, and scientific publications of Kunz and his co-workers from that period reflect the results of the work done and reported under US Army contracts. Furthermore, in 1971 Kunz became a professor of virology and received his own institute, separate from the Institute of Hygiene.^63^

In the 1967 report Kunz spoke of his first (unsuccessful) attempts to immunize mice against TBE — but until 1972 the reports did not mention a vaccine for humans. In 1972, however, Kunz reported that he was expecting to try the first batches of the new inactivated TBE vaccine that should be sent to him from “a laboratory in Great Britain.”^64^ What was this vaccine and where did it come from?

## From the British to the Austrian Vaccine

In the existing public narrative in Austria, the vaccine against TBE was developed by Christian Kunz and was a logical continuation of the previous Austrian work on TBE starting with Hans Schneider in the 1930s and the local response to the local Austrian disease. The cooperation with Great Britain is mentioned in some scientific accounts, including by Kunz himself, but only very briefly.^65^ However, archival sources, including those that have only recently been declassified, reveal a somewhat different story and show that at the early stages of vaccine development Austrian scientists played a more supportive role.

The laboratory in Great Britain that produced the TBE vaccine that Kunz mentioned in his 1972 report for the US Army was the Microbiological Research Establishment (MRE) Porton Down, the UK’s main facility for military research in microbiology that belonged to the Ministry of Defense. Microbiology at Porton Down started in the 1940s as a top secret endeavor to study the possibilities of biological warfare but by the 1960s, after Britain stopped its offensive programs, its research moved to defensive measures against pathogens and especially vaccine development. The virology section was established at Porton in 1953 and initially concentrated on the pox virus. Following the global explosion of interest in arbovirology, in 1963-1964 the MRE Porton Down set up the Arthropod-Borne Virus Epidemiology Unit and the focus switched to arbovirus research and especially Japanese encephalitis virus and louping-ill virus. The creation in the 1970s of a special high containment unit for arbovirus research endowed the complex with the technical infrastructure to safely handle some of the world’s most dangerous pathogens.^66^

In 1970, MRE Porton Down started research on the pathogenesis of viral encephalitis and particularly the role of immune response, the active and passive immunity and immunosuppression. The experiments involved viruses of the TBE complex and among them especially the louping-ill virus, a tick-borne pathogen of sheep that is endemic in the UK and can occasionally cause dangerous human disease. The outcome of this research was the development of the human vaccine against louping-ill that went to small-scale human trials in 1971. Tick-borne encephalitis virus is closely related to that of louping-ill, and the attempts to develop a vaccine against it were, as the reports of the MRE Porton Down stated, a “logical sequel” of the work with louping-ill.^67^

It is unclear how exactly the cooperation between the MRE Porton Down and Kunz came about. On the British side, the key person involved in TBE vaccine development was veterinary microbiologist James Keppie whose specialization was the pathogenicity of zoonotic microbial disease agents and, from the turn of the 1970s, also vaccine production. However, I could not identify any direct communication between Keppie and Kunz. The correspondence related to the TBE vaccine production went through R.J.C. Harris, the Director of MRE Porton Down, and did not mention any other contacts or cooperation beyond the TBE vaccine. It is possible that some role in bringing the two sides together was played by Norman Finter, the Head of Virology at the Wellcome Research Laboratories in the UK, who seemed to act as an intermediary in the correspondence and negotiations between Kunz and Porton Down. There could have also been more personal connections as Finter had a Viennese wife.^68^

In 1971, the MRE Porton Down began the work on the TBE vaccine based on the Neudörfl strain of the Central European TBE virus supplied by Kunz. By 1972 the first batches of the experimental vaccine were produced. The vaccine was based on the highly concentrated virus culture grown in chick-embryo cells suspended in Medium 199 (a synthetic media widely used since the 1950s to grow animal cells), which was then purified by absorption-elution procedure with hydroxylapatite, inactivated with formalin, dialyzed, and pooled. The antigenicity of the vaccine was then tested on mice and monkeys. At that stage the MRE did not test the vaccine on humans but instead outsourced the human trials to Kunz in Austria.^69^ Just like Elizaveta Levkovich and Galina Zorina-Nikolaieva in 1939, Kunz first tested the new TBE vaccine on himself and his co-worker Hans Hoffmann. It is revealing to compare the human trials of the TBE vaccine in the Soviet Union and Austria, even though they were conducted in very different scientific and political circumstances and were dealing with TBEV subtypes of different severity.

In a 1973 trial by Kunz, the new TBE vaccine was tested on 81 seronegative volunteers from Austria by hemagglutination inhibition assay that showed that 76% of them after the first dose, 96% after the second, and 98% after the third developed antibodies against the disease. At this stage, the protection against the disease in real-life situation was not tested, and neither the trial’s published materials nor Kunz’s earlier internal report to the public health authorities in Austria mentioned the gender and age of the vaccinees. There is no indication that scientists at Porton Down had any influence over the design of the human trial but they were quick to recognize its results as a proof of their success. That very limited trial allowed the MRE to report the development of the efficient vaccine against TBE and the Austrian side to move to public vaccination programs against TBE in 1974.^70^

The accounts of the 1973 Austrian trial clearly spoke of volunteers. The following year, however, the vaccine was given already to 5000 people, mostly forestry workers from Lower Austria, who were then also observed. They were no longer described as volunteers and, in fact, had to pay for the vaccine (although in some cases the costs were covered by their health insurance if TBE was considered an occupational hazard). To prove the effectiveness of the vaccine, about one-fifth of the vaccinees were later checked for their antibodies but, as there was no mention of their seropositivity having been controlled before the vaccination, it was unclear whether this immunity was a result of the vaccine or of a natural infection from that or previous years — a likely possibility in a highly-exposed group facing a virus subtype with which the majority of infections pass without symptoms. There was no control group, unlike in the Soviet trials in the 1930s and in the 1960s, and no attention was paid to age, gender or exposure differences. The only proof that the vaccine offered effective protection against TBE was that none of the vaccinees developed a visible clinical infection that year and none of the 296 persons hospitalized with TBE in Austria in 1974 had been vaccinated. In 1975, already 30,000 persons received the new vaccine, including children from the age of three.^71^

There are two important aspects about this stage of the British-Austrian vaccine collaboration. First, the MRE’s experiments with TBE and TBE vaccine development as well as the research on viral encephalitis more generally were classified as a part of the defense and not of the rapidly growing MRE civil program. As such it did not get any external evaluation and the first publications about it appeared only in 1976, when public vaccination campaigns were already underway.

Secondly, at that stage the vaccine was not described as the invention of Kunz. On the contrary, all actors, including Kunz himself, emphasized the British agency and described it as the “MRE vaccine,” “vaccine from the British laboratory/producer,” and the production process as “Dr. Keppie’s work.” The role of Kunz in this collaboration appeared to be limited to supplying the virus strain and organizing the human trials.^72^ It was only in the second half of the 1970s that the vaccine began changing its identity from the “British” to the “Austrian.”

In the 1970s TBE was not endemic on the British Isles so the new vaccine had little public health and commercial potential in the UK. In Austria, where TBE was endemic and where industrial and increasingly also recreational use of forests meant high exposure to tick bites, the possibilities for commercial application of the vaccine were substantial. TBE was a relatively rare disease, and although in most cases the infection passes without symptoms and there is no possibility of human-to-human transmission, the public demand for the vaccine was huge and the project enjoyed support at the highest governmental level. Despite the very limited medical studies of the vaccine, the political interest in it in Austria was such that already in summer 1974 the president of Austria, Rudolf Kirchschläger, discussed with the Minister of Public Health and Environment Ingrid Leodolter the possibility of making the TBE vaccination obligatory for school children in Austria — the suggestion that she prudently rejected. However, Leodolter herself was ready to advertise TBE vaccination for school children, and the only factor preventing it was not safety concerns but the production limitations at Porton Down.^73^

Porton Down also recognized the commercial prospects of the huge public demand for the vaccine in Austria. Throughout the 1970s MRE became much more visible in the civil healthcare sector, and for-profit activities were used to compensate for the defense cuts. In 1975, TBE vaccine production moved from the defense to the civil program at the MRE and eventually turned one of its key elements. The MRE animal isolation facility in Allington was converted into a unit for vaccine manufacturing. Scientists at Porton Down also explored ways of optimizing the manufacturing process to adjust it to mass production.^74^ In particular, they experimented with various ways of purification and already in 1975 suggested a new technique of purification through zonal ultracentrifugation, which they believed to be more efficient but also more expensive and therefore warranted only by the sufficient demand for the product.^75^

Although MRE remained the main manufacturer of the vaccine, from 1975 the vaccine production was divided between Porton Down and the Austrian company Immuno AG. The vaccine was produced in Britain and supplied in bulk, but the ampule filling, distribution, and marketing were done in Austria. During the winter season of 1977-1978, Porton Down supplied 329,000 doses of the vaccine. The MRE was not ready to undergo the full licensing process for the vaccine according to the British apparently more rigorous standards, and after the negotiations with Kunz, Immuno, and the Austrian authorities, the product liability was assumed by the Austrian side.^76^

At this point, however, the remarkably smooth civil conversion and commercialization of the vaccine encountered a problem. The rapid expansion of the immunization program in Austria with little preliminary research and control revealed high reactogenicity of the vaccine, especially among children, with severe side effects, including fainting and convulsions. Following the frequent reports of severe side effects early in 1978, the vaccination programs in Austria were urgently halted. When appearing on the Austrian media, Kunz claimed that these collapse cases had nothing to do with the vaccine but instead were related to the influenza epidemic and the fears of injection among the vaccinees.^77^ The investigation at the MRE, however, revealed that the unwanted reactions to the vaccine were caused by the contamination of the vaccine with the residual cell material. To avoid this problem, MRE scientists proposed the modification of the purification process and the introduction of zonal ultracentrifugation that they had suggested already three years earlier. The experimental batches of this new version of the vaccine were again designed and produced at Porton Down and then sent for tests in Austria the same year.^78^ During the trial, vaccine batches from Porton were tested on 70-134 adults and 38-112 children between 3 and 14 years of age (the numbers of vaccinees observed after each dose differed). The trial showed a somewhat lower frequency of severe side reactions. The trial results did not describe the vaccinees as volunteers and it is unclear whether the participants were informed that they were receiving a new version of the vaccine that had not been tested on humans before. Although the number of participants was small, Kunz and his co-workers concluded that the new vaccine represented “an excellent means for the prophylaxis of TBE.”^79^ In 1981, Austria started a nationwide vaccination campaign against TBE using this vaccine.

Ironically, the 1978 vaccine scandal contributed to its further “austrianization.” In light of the concerns over the vaccine safety, the relative laxity of medication regulations in Austria, and the apparent success of the ultracentrifuge-prepared vaccine, Porton Down decided to halt the production of the vaccine by the old method but instead supply concentrated virus supernatant material which would be centrifuged already in Austria and would be for Austrian use only.^80^ In December 1978, Immuno AG submitted a patent application for the preparation of the TBE vaccine through centrifugation (approved in 1980). The list of inventors included only Kunz and his colleagues. No British scientist was mentioned.^81^ The modified vaccine officially became an Austrian invention. Although Kunz and his colleagues acknowledged the cooperation with MRE and specifically James Keppie in the text of their publications about the vaccine, he was never included as a co-author.^82^ When in 1986, the Austrian Chamber of Pharmacists held a press conference in connection with the five-year anniversary of the vaccination campaign, the British role in the development of the vaccine was completely omitted from the report, and Kunz appeared as the only inventor.^83^

The cooperation with the MRE, however, continued. When Austria moved to a nationwide immunization campaign against TBE, Immuno AG financed the construction at Porton Down of the dedicated unit for the production of the TBE vaccine. The unit was owned by Immuno but the technical expertise, manpower, and equipment were provided by Porton Down. Several years later Immuno transferred the production to Austria, completing the “austrianization” of the vaccine. The Immuno building was purchased by the MRE successor, the Center for Applied Microbiology Research, for a symbolic sum of one pound. Even though Immuno itself ceased to exist (it was purchased by Baxter in 1996), the building kept the name of the Austrian company until the twenty-first century as a reminder of the transnational cooperation in the TBE vaccine production.^84^

The TBE vaccination campaign in Austria, supported by intensive marketing, was extremely successful. Although the vaccine was relatively expensive and not covered by public insurance, by 1985 more than 30% and by 1994 more than 70% of the Austrian population received the shot,^85^ proving the safety of the vaccine and validating it for international use. When in the 1990s cases of TBE started to grow, possibly due to social and environmental transformations connected to post-socialist transition, the Immuno vaccine was approved and used in public health programs in other countries of Central and Eastern Europe.^86^

## Conclusion

To conclude, I would like to highlight five points discussed in the article above. First, from the very beginning the research on the tick-borne encephalitis virus and the development of the TBE vaccine in the Soviet Union were inseparable from the political and military considerations connected to the long Second World War and the Stalinist colonization of the Far East through involuntary resettlement and forced labor. Those factors determined the visibility and epidemiology of TBE and its perception as extremely dangerous, explained the interest of the central authorities in Moscow in supporting this project, shaped the process of vaccine development and validation, including human trials, and the interpretation of their results. Despite the importance of the military agenda for the initial TBE research, the development of the TBE vaccine was a civil project, and its results were quickly shared with the international community both through publications and bilateral cooperations throughout the 1940s. Importantly, the TBE expeditions of the 1930s had profound consequences for Soviet biomedicine: they attracted attention, infrastructural support and funding to the new field of virology, propelled the careers of scientists who would define that field for years to come, and turned research on arthropod-borne and neurotropic viruses into a dominant field of virology in the Soviet Union and eventually also in the Eastern bloc countries.

Second, from the 1940s onwards and especially with de-stalinization, TBE research became the subject of intensive scientific exchange. This exchange started already during WWII and later developed both within the Eastern bloc and across the Iron Curtain, with the Soviet and Central European, especially Czechoslovak, scientists playing an active role. This process encompassed a variety of forms, such as study trips and individual visiting fellowships, conferences, publication, and virus strain exchange, and influenced scientists and research projects on both sides. This cooperation intensified with time and by the early 1960s emerged into multilateral international projects which in turn led to the standardization of nomenclature, research, and diagnostic techniques and the establishment of the network of reference centers in arbovirology. Because of this cooperation, the Soviet TBE research and vaccine development in the 1960s turned into a much more international project than it had been in the 1930s, despite strong personal and institutional continuities, and has to be viewed in the context of global scientific processes rather than within the narrow national framework.

Third, parallel to this open and civil scientific internationalism, TBE emerged as a topic of military interest on behalf of the Western allies and the fears of weaponization. As a result, the research on this disease and prevention mechanisms against it in the West considerably profited from the military funding and infrastructures. Furthermore, those military interests also fostered transnational cooperation that had a profound impact on the TBE research in officially neutral countries such as Austria.

The last two points refer more specifically to the Austrian case. I have shown in the article that this transnational scientific research was crucial for the TBE study in Austria — the major point that would be missed when interpreting it solely through the lens of the national scientific school. Drawing a direct link between the description of epidemic meningitis by Hans Schneider in the 1930s and the research of Hans Moritsch in the 1950s to the vaccination campaigns in the 1970s and 1980s omits not only the many etiological and diagnostic uncertainties of the 1930s-1950s but also fundamental steps made by the Soviet, Czechoslovak, and Dutch scientists who isolated the virus, connected the disease to the vector, described the transmission routes, identified the virus that caused the meningoencephalitis outbreaks in Austria, and linked it to TBEV complex and other outbreaks in the region. Furthermore, the narrow national interpretation completely ignores the importance of military agenda, foreign infrastructure, and financial support, as well as the Cold War geopolitics, to the development of Austrian TBE research and virology more broadly.

Finally, this argument is also valid for the development of the TBE vaccine that would be used for the Austrian mass immunization campaigns. The development of that vaccine did not happen in isolation from the Soviet vaccines that were directly studied — even if eventually rejected — by the Austrian scientists. Most importantly, the development of the Austrian vaccine, which so often is credited to Christian Kunz alone, was in fact a British-Austrian project that, like the Austrian TBE research in general, was directly influenced by Cold War agendas and military concerns. Perhaps somewhat surprisingly, although the Soviet TBE vaccine was not a military project, the Austro-British vaccine was. It was developed within the walls of the institution that belonged to the British Ministry of Defense and was classified as a part of the defense program. Importantly, it was the scientists at Porton Down, in particular, James Keppie, who played a crucial role in the development of both the older and the newer version of the TBE vaccine and they deserve greater credit than they have received so far. Although the vaccine was later “austrianized” first through limited human trials and then through the relocation of production to Austria and mass usage in the 1980s, it is worth remembering that it was a product of a transnational scientific endeavor.

## Footnotes

1 For a recent review, see Daniel Ruzek, Tatjana Avšič Županc, Johannes Borde, Ales Chrdle, Ludek Eyer, Galina Karganova, Ivan Kholodilov, Nataša Knap, Liubov Kozlovskaya, Andrey Matveev, Andrew D. Miller, Dmitry I. Osolodkin, Anna K. Ӧverby, Nina Tikunova, Sergey Tkachev, Joanna Zajkowska, “Tick-Borne Encephalitis in Europe and Russia: Review of Pathogenesis, Clinical Features, Therapy, and Vaccines,” *Antiviral Research* 164 (2019): 23-51.

2 “Vaccines Against Tick-Borne Encephalitis: WHO Position Paper,” *WHO Weekly Epidemiological Record* 24 (10 June 2011); *Global Vector Control Response* (Geneva: World Health Organization, 2017), 41; see also current information online https://www.who.int/health-topics/tick-borne-encephalitis#tab=tab_1.

3 “Vaccines Against Tick-Borne Encephalitis,” 246-250; Mareike Kubinski, Jana Beicht, Thomas Gerlach, Asisa Volz, Gerd Sutter, Guus F. Rimmelzwaan, “Tick-Borne Encephalitis Virus: A Quest for Better Vaccines against a Virus on the Rise,” *Vaccines* 8: 3 (2020): 451.

4 Christian Kunz, “TBE Vaccination and the Austrian Experience,” *Vaccine* 21 (2003): 50-55; J. Süss, “Tick-borne Encephalitis in Europe and Beyond – the Epidemiological Situation as of 2007,” *Eurosurveillance* 13: 26 (2008), https://doi.org/10.2807/ese.13.26.18916-en.

5 In Russia, this narrative is visible in a considerable medical-historical research, including several collections of biographical documents, which were then used in public history publications and documentaries. See for example, S.G. Drozdov, ed., *Vospominaniia o Mikhaile Petroviche Chumakove* (Moscow: Institut poliomielita i virusnykh entsefalitov im. Chumakova, 1999); V.V. Pogodina, ed., *Vospominaniia o Elizavete Nikolaievne Levkovich* (Moscow: Institut poliomielita i virusnykh entsefalitov im. Chumakova, 2001); L.L. Kiselev and E.S. Levina, *Lev Aleksandrovich Zil’ber, 1894-1966: zhizn’ v nauke* (Moscow: Nauka, 2005); E.S. Levina, “Pervyie virusologi i meditsinskaia virusologiia v SSSR 1930-kh godov,” *Istoriko-biologicheskie issledovaniia* 2 (2010): 10-50. In Austria, no comparable historical work has been done but the public narrative can be seen for example in the following 2016 article in *Der Standard* (one of Austria’s leading newspapers), entitled “Tick-Borne Encephalitis Vaccine: An Austrian Invention,” https://www.derstandard.at/story/2000032266296/fsme-impfstoff-eine-oesterreichische-erfindung.

6 John Krige, *American Hegemony and the Postwar Reconstruction of Science in Europe* (Cambridge, MA: MIT Press, 2006); Naomi Oreskes and John Krige, *Science and Technology in the Global Cold War* (Cambridge, MA: MIT Press, 2014); Soraya de Chadarevian and Harmke Kamminga, *Molecularizing Biology and Medicine: New Practices and Alliances, 1910s-1970s* (Amsterdam: Harwood Academic, 1998); Toby A. Appel, *Shaping Biology: The National Science Foundation and American Biological Research, 1952-1975* (Baltimore: Johns Hopkins University Press, 2000); Soraya de Chadarevian, *Designs for Life: Molecular Biology After World War II* (Cambridge: Cambridge University Press, 2002); Angela Creager, *The Life of a Virus: Tobacco Mosaic Virus as an Experimental Model, 1930-1965* (Chicago: University of Chicago Press, 2002); Angela Creager, *Life Atomic: A History of Radioisotopes in Science and Medicine* (Chicago: University of Chicago Press, 2013); Ellen Leopold, *Under the Radar: Cancer and the Cold War* (New Brunswick, NJ: Rutgers University Press, 2009); Jacob Hamblin, *Arming Mother Nature: The Birth of Catastrophic Environmentalism* (Oxford: Oxford University Press, 2013).

7 Milton Leitenberg and Raymond A. Zilinskas, *The Soviet Biological Weapons Program: A History* (Cambridge, MA: Harvard University Press, 2012); Anthony Rimmington, *Stalin’s Secret Weapon: The Origins of Soviet Biological Warfare* (London: Hurst & Company, 2018); Nikolai Krementsov, *The Cure: A Story of Cancer and Politics from the Annals of the Cold War* (Chicago: University of Chicago Press, 2002); Anna Geltser, “Surrogate Epistemology: The Transition from Soviet to Russian Biomedicine” (Ph.D. diss., Cornell University, 2012).

8 Dóra Vargha, *Polio Across the Iron Curtain: Hungary’s Cold War with an Epidemic* (Cambridge: Cambridge University Press, 2018); Johannes Feichtinger and Heidemarie Uhl, ed., *Die Akademien der Wissenschaften in Zentraleuropa im Kalten Krieg* (Vienna: Verlag der Österreichischen Akademie der Wissenschaften, 2018); Sławomir Łotysz, “Knowledge as Aid: Locals Experts, International Health Organizations and Building the First Czechoslovak Penicillin Factory, 1944–9,” in *Europe’s Internationalists: Rethinking the History of Internationalism*, ed. Jessica Reinisch and David Brydan (London: Bloomsbury, 2021), 140-157.

9 Levina, “Pervyie virusologi.” For an English-language overview of the history of TBE in the Far East before the arrival of Zilber’s expedition, see Lisa Walker, “Public Health in the Soviet Periphery: The Case of Tick-Borne Encephalitis, 1932-1937,” NCEEER Working Paper, University of Washington, 2011. An English-language (rather laudatory) account of Zilber’s life and research, written by his student and two sons, can be found at Lev L. Kisselev, Gary I. Abelev and Feodor Kisseljov, “Lev Zilber: The Personality and the Scientist,” *Advances of Cancer Research* 59 (1992): 1-40. On the history of Soviet science and specifically biology in the 1930s, see Nikolai Krementsov, *Stalinist Science* (Princeton: Princeton University Press, 1997), chapters 2 and 3.

10 On Evgeny Pavlosvky and the importance of his work for Soviet public health, see Susan Jones and Anna Amramina, “Entangled Histories of Plague Ecology in Russia and the USSR,” *History and Philosophy of Life Science* 40: 49 (2018), doi:10.1007/s40656-018-0220-3. On the international significance of Pavlovsky’s work, see Telford Work, “Tick-Borne Viruses: A Review of an Arthropod-borne Virus Problem of Growing Importance in the Tropics,” *Bulletin of the WHO* 29 (1963): 59-74, 61; William Reisen, “Landscape Epidemiology of Vector-Borne Diseases,” *Annual Review of Entomology* 55 (2010): 461-483, 462; Jakob Zinsstag, David Waltner-Toews, and Marcel Tanner, “Theoretical Issues of One Health” in *One Health: The Theory and Practice of Integrated Health Approaches*, ed. Jakob Zinsstag, Esther Schelling, Lisa Crump, Maxine Whittaker, Marcel Tanner, Craig Stephen (Wallingford: CABI Publishing, 2021), 16-25, 21. An English translation of Pavlovsky’s work was published as E.N. Pavlovskii, *Natural Nidality of Transmissible Diseases: With Special Reference to the Landscape Epidemiology of Zooanthroponoses* (Urbana: University of Illinois Press, 1966).

11 N.I. Grascenkov, “Tick-borne Encephalitis in the USSR,” *Bulletin of the WHO* 30 (1964): 187-196.

12 Süss, “Tick-borne Encephalitis in Europe and Beyond”; Ruzek et al., “Tick-Borne Encephalitis in Europe and Russia.”

13 N.M. Koliasnikova et al., “Evolutsiia kleshchevogo encephalita za 80-letinii period: osnovnyie proiavleniia, veroiatnyie prichiny,” *Epidemiologiia i vaktsinoprofilaktika* 19 (2020): 78-88.

14 Pogodina, *Vospominaniia o Elizavete Nikolaievne Levkovich*, 81-95.

15 Rimmington, *Stalin’s Secret Weapon*, 3-10.

16 On the feminization of Soviet science and especially natural sciences and medicine in the interwar period, see E.A. Dolgova and E.A. Streltsova, ””Dobro pozhalovat’ v klub”: Polozhenie zhenshchin v sovetskoi nauke v 1920-e gg.,” *Sotsiologicheskiie issledovaniia* 2 (2019): 97-107; Alexei Kojevnikov, “The Phenomenon of Soviet Science,” *Osiris* 23 (2008), https://doi.org/10.1086/591871.

17 Levina, “Pervyie virusolog*i*,” 37; Pogodina, *Vospominaniia o Elizavete Nikolaievne Levkovich*, 81-95.

18 A.A. Smorodintseff, N.W. Kagan, E.N. Levkovitsch et al., ”Experimenteller und epidemiologischer Beitrag zur aktiven Immunisierung gegen die Frühling-Sommer-Zeckenencephalitis,”*Archiv f Virusforschung* 2 (1941): 1–25. The memoir of A.N. Shapoval is published in Pogodina, *Vospominaniia o Elizavete Nikolaievne Levkovich*, 47-59, the cited numbers are from 57.

19 Levina, “Pervyie virusolog*i*,” 37; Pogodina, *Vospominaniia o Elizavete Nikolaievne Levkovich*, 47-59; V.V. Pogodina et al., “75-letiie otkrytiia kleshchevogo entsefalita: sravneniie rannikh (1937-1945) i sovremennykh shtammov,” *Voprosy virusologii* 1 (2012): 66-75.

20 Golfo Alexopoulos, “Medical Research in Stalin’s Gulag,” *Bulletin of the History of Medicine* 90 (2016): 363-393; Dan Healey, “Lives in the Balance: Weak and Disabled Prisoners and the Biopolitics of the Gulag,” *Kritika: Explorations in Russian and Eurasian History* 16 (2015): 527-556. See also B. A. Nakhapetov, *Ocherki istorii sanitarnoi sluzhby GULAGa* (Moscow: ROSSPEN, 2009).

21 For the discussion of the role of WWII in the cooperation between Soviet and Western scientists and the examples of such cooperation in the fields of microbiology and cancer research, see Nikolai Krementsov, *The Cure: A Story of Cancer and Politics From the Annals of the Cold War* (Chicago: University of Chicago Press, 2002).

22 A.A. Smorodintseff, “The Spring-Summer Tick-Borne Encephalitis,” *Archiv f Virusforschung* 1 (1940): 468–480; M.P. Chumakov and N.A. Seitlenok, “Tick-Borne Encephalitis in the European Part of the USSR and Siberia,” *Science* 92: 2386 (1940): 263-264; Smorodintseff, Kagan, Levkovitsch, “Experimenteller und epidemiologischer Beitrag.”

23 Jordi Casalas and Peter Olitzky, “Enduring Immunity Following the Vaccination of Mice with Formalin-Inactivated Virus of Russian Spring-Summer (Far-Eastern, Tick-Borne) Encephalitis,” *Journal of Experimental Medicine* 82 (1945): 431–443.

24 See for example, 1945 correspondence between Smoroditsev and Smadel from the Albert Sabin Digital Archives at the University of Cincinnati: http://hdl.handle.net/2374.UC/665195; http://hdl.handle.net/2374.UC/665196; https://drc.libraries.uc.edu/handle/2374.UC/678592; http://hdl.handle.net/2374.UC/667075. The Soviet-American scientific exchange during WWII is discussed in Saul Benison, “International Medical Cooperation: Dr. Albert Sabin, Live Poliovirus and the Soviets,” *Bulletin of the History of Medicine* 56 (1982): 462-465.

25 Frantisek Gallia, Josef Rampas, and Ludvik Hollender, “Laboratorni infekce encefelytickym virem,” *Časopis lékařů českých* 88: 9 (1949): 224-225; Erika Richling, “Virus Meningo-Encephalitis in Austria: Epidemiological Features,” *Bulletin of the World Health Organization* 12 (1955): 521-534.

26 For the discussion of the environmental impact of WWII and post-war reconstruction in Europe on the tick vectors see Harry Hoogstraal, “The Epidemiology of Tick-Borne Crimean-Congo Hemorrhagic Fever in Asia, Europe, and Africa,” *Journal of Medical Entomology* 15 (1979): 307-417, 343-345. On the importance of the nutritional state and general health status for the severity of TBE symptoms, see Ruzek et al., “Tick-Borne Encephalitis in Europe and Russia.”

27 Gallia, Rampas, and Hollender, “Laboratorne infekce”; Josef Rampas and Frantisek Gallia, “Isolaca viru encefalitidy z klistat Ixodes ricinus,” *Časopis lékařů českých* 88: 41 (1949): 1179-1180.

28 Work, “Tick-borne Viruses,” 63-64; Dionyz Blaskovic, “The Public Health Importance of Tick-borne Encephalitis in Europe,” *Bulletin of the World Health Organization* 36: suppl 1 (1967): 5-13.

29 Work, “Tick-borne Viruses,” 64; Blaskovic, “Public Health Importance.” The records concerning the fellowship of Dionyz Blaskovic are located at the Rockefeller Foundation Archives, 712.E.

30 On the organizational development and science internationalism in Czechoslovakia, see Victor McElheny, “Biological Research in Czechoslovakia,” *Science* 145: 3634 (1964): 799-802; Doubravka Olšáková, “The International Biological Program in Eastern Europe: Science Diplomacy, Comecon and the Beginnings of Ecology in Czechoslovakia,” *Environment and History* 24 (2018): 543-567; Adam Hudek and Dusan Kovac, “Neue Wissenschaft für die Neue Gesellschaft. Die Transformation der Slowakischen Akademie der Wissenschaften in den Jahren 1945-1967,” in *Die Akademien der Wissenschaften in Zentraleuropa*, 199-227.

31 Helena Libikova, ed., *Biology of the Viruses of the Tick-Borne Encephalitis Complex: Proceeding of a Symposium held in Smolenice, Oct. 11-14 1960* (Prague: Czechoslovak Academy of Sciences, 1962).

32
*Arthropod-borne Viruses: Report of the Study Group* (Geneva: World Health Organization, 1961); A.C. Sáenz, ”The WHO System of Arthropod-Borne Virus Reference Centres,” *Bulletin of the World Health Organization* 35 (1966): 93-94; Work, “Tick-Borne Viruses,” 70-71.

33
*Arboviruses and Human Disease: Report of a Scientific Group* (Geneva: World Health Organization, 1967), 6.

34 Delphine Clarke, “Further Studies on Antigenic Relationships Among the Viruses of the Group B Tick-Borne Complex,” *Bulletin of the World Health Organization* 31 (1964): 45-56.

35 Work, “Tick-Borne Viruses,” 68.

36 Pogodina, *Vospominaniia o Elizavete Nikolaievne Levkovich*, 51-52.

37 Benison, “International Medical Cooperation,” 465-469; Vargha, *Polio Across the Iron Curtain,* 153-159.

38 Work, “Tick-Borne Viruses,” 62; *Arboviruses and Human Disease*, 4.

39 M.P. Chumakov et al., “Sravnitel’noie izucheniie epidemiologicheskoi effektivnosti privivok kul’tural’noi i mozgovoi vaktsiny protiv kleshchevogo entsefalita,” *Voprosy virusologii* 3 (1963): 307-315; Lev Goldfarb, “Six Lectures on the Prevention of Encephalitis Epidemics in Siberia,” *Siberian Research* 1 (2020): 61-99, 73-74.

40 The reports of Jakob Brody are available at the Albert Sabin Digital Archives, http://hdl.handle.net/2374.UC/678557; see also Goldfarb, “Six Lectures,” 67.

41 Goldfarb, “Six Lectures,” 73-74; Work, “Tick-Borne Viruses,” 69-70; G.P. Bilalova et al., “Istoriia proizvodstva vaktsin dlia profilaktiki kleshchevogo entsefalita v gorode Tomske: ot mozgovoi vaktsiny do vaktsiny Encevir,” *Bulleten SO RAN* 4: 126 (2007): 105-110.

42 OeStA [Austrian State Archive], 51756/75.

43 Blaskovic, “Public Health Importance,” 10-12.

44 Hans Schneider, “Über epidemische akute ‘Meningitis serosa,’” *Wien klin Wochenschr* 44 (1931): 350–352.

45 This point was made, for example, in the presentation of Gerhard Dobler at the ISW-TBE meeting in Vienna, 9-10 September 2021.

46 In particular, Kozhevnikov epilepsy, described by A. Ia. Kozhevnikov in 1894, was linked to infectious encephalitis by L.I. Omorokov in 1927 on the basis of this research in Siberia. See L. Omorokov, ”Die Kojewnikoffsche Epilepsie in Sibirien,” *Z Ges Neurol Psych* 107 (1927): 487–496; Ruzek et al., “Tick-Borne Encephalitis.” For the discussion of the history of Kozhevnikov epilepsy and its links to TBE, see Alla A. Vein and W. van Emde Boas, “Kozhevnikov Epilepsy: The Disease and its Eponym,” *Epilepsia* 52 (2011): 212-218.

47 Richling, “Virus Meningo-Encephalitis in Austria.”

48 J.D. Verlinde et al., “Virus Meningo-Encephalitis in Austria: Pathogenic and Immunological Properties of the Virus,” *Bulletin of the World Health Organization* 12 (1955): 565-579.

49 H. Moritsch and J. Krausler, “Die endemische Frühsommer-Meningo-Encephalo-Myelitis,” *Wien klin Wochenschr* 49 (1957): 921–926, 952–956, 965–970; H. Moritsch and J. Krausler, “Die Frühsommer-Meningo-Enzephalitis in Niederösterreich 1956-1958,” *Deutsche Medizinische Wochenschrift* 84 (1959): 1934-1939.

50 Hans Moritsch, “Die Encephalitisviren,” in *Klinische Probleme der Poliomyelitis und verwandter Viruskrankheiten*, ed. F. Wöhler and Oskar Vivell (Berlin: Springer, 1961), 12-20; Hans Moritsch, “Die Arbo-viren,” in *Virus- und Rickettsieninfektionen des Menschen*, ed. Richard Haas and Oskar Vivell (Berlin: Springer, 1965), 412-494.

51 Ingrid Arias, “Die Medizinische Fakultät von 1945 bis 1955: Provinzialisierung oder Anschluss an die westliche Wissenschaft,” in *Zukunft mit Altlasten: Die Universität Wien 1945 bis 1955*, ed. Margarete Grandner et al. (Innsbruck: Studien Verlag, 2005), 68-88, 82-83; Heinz Flamm, “1908–2008 – Hundert Jahre Hygiene-Institut der Universität Wien,” *Wien Klin Wochenschr* 120 (2008): 571–580, 578-579.

52 Olšáková, “International Biological Program in Eastern Europe*”*; George Garland, “Decisions Taken at the ICSU Executive Board and General Assembly Meetings of Special Interest to IUGG Vienna, November 1963,” *Transactions, American Geophysical Union* 45 (1964): 137–140.

53 Jan Vilcek, *Love and Science: A Memoir* (New York: Seven Sisters Press, 2016), 142-147.

54 Army Research Office. *Army Research Task Summary, Fiscal Year 1960*. Volume 1. *Medical, Biological, and Social and Behavioral Sciences* (Washington, DC: Department of the Army, Office of the Chief of Research and Development [1960]), 147, contract number DA 91-591-EUC-1379.

55 Leitenberg and Zilinskas, *Soviet Biological Weapons Program,* 108-110.

56 US National Archives, Records of the CIA 1894-2002, NIE 11-6-64, 26 Aug 1964, *Soviet Capabilities and Intentions with Respect to Biological Warfare*, https://catalog.archives.gov/id/7327228.

57 Krige, *American Hegemony*, 3.

58 I have identified research contracts for 1960, 1964 and continuously for 1966-1972: Defense Technical Information Center, AD0696524 https://apps.dtic.mil/sti/citations/AD0696524; AD0751205 https://apps.dtic.mil/sti/citations/AD0751205; AD0816540 https://apps.dtic.mil/sti/citations/AD0816540; Department of Defense Appropriations for 1966. Hearings Before a Subcommittee of the Committee of the Appropriations, House of Representatives, Eighty-Ninth Congress, 1965, Part 4, 290-291.

59 The exact cause of death is, however, unclear. American sources and Vilcek say that it was TBE but Moritsch’s successor at the Hygiene Institute Heinz Flamm claimed that the encephalitis was caused by the herpes virus. See Vilcek, *Love and Science*, 146; Heinz Flamm, “1908–2008 – Hundert Jahre Hygiene-Institut der Universität Wien,” *Wien Klin Wochenschr* 120 (2008): 571–580, 579.

60 Department of Defense Appropriations for 1967. Hearings Before a Subcommittee of the Committee of the Appropriations, House of Representatives, Eighty-Ninth Congress, 1966, Part 4, 224.

61 Hans Moritsch, “Entwicklung der Virusforschung in Österreich,” in *Aufgaben der Universität Wien in Gegenwart und Zukunft* (Vienna: Verlag der Österreichischen Hochschulzeitung, 1965), 129-133.

62 Vilcek, *Love and Science*, 150; the documents concerning Christian Kunz’s fellowship in the US are located at the Rockefeller Foundation Archives, 705.E.

63 Franz X. Heinz, “Obituary for Christian Kunz, 1927–2020,” *Wien Klin Wochenschr* 132 (2020): 410–411; see also Ch. Kunz und H. Hofmann, “Die Beeinflussung der experimentellen Frühsommer-Meningoenzephalitis-(FSME-)Virusinfektion durch die Interferon induzierende Substanz Po]y I:C.,” *Zbl. Bakt.*, I.Abt. Orig. 211 (1969): 2700273; H. Hofmann and C. Kunz, “The Protective Effect of the Interferon Inducers Tilorone Hydrochloride and Poly I:C on Experimental Tick-borne Encephalitis in Mice,” *Archiv f Virusforschung* 37 (1972): 262–266.

64 Defense Technical Information Center, AD0751205, https://apps.dtic.mil/sti/citations/AD0751205; AD0816540, https://apps.dtic.mil/sti/citations/AD0816540.

65 Kunz, “TBE Vaccination.”

66 Peter Hammond and Gradon Carter, *From Biological Warfare to Healthcare: Porton Down, 1940-2000* (Basingstoke: Palgrave, 2002), 3, 228; Sophie Smither and Mark Lever, “A Review of Filovirus Work and Facilities at The Defence Science and Technology Laboratory Porton Down,” *Viruses* 4 (2012): 1305–1317, 1306.

67 The National Archives (UK), DEFE 55/360:73; DEFE 55/160:48-50; DEFE 55/160:70; DEFE 55/161:45-50.

68 OeStA, 51.176/74. On Keppie and his research see, James Keppie, “Mechanisms of Bacterial Pathogenicity and Tissue Localization,” Dr. Sc. Dissertation, University of Edinburgh, 1965; H. Smith, “The Mounting Interest in Bacterial and Viral Pathogenicity,” *Annual Review of Microbiology* 43(1989): 1-22, 2. On Finter see, Derek C. Burke and Tony Meager, “Norman B. Finter, MD (1924-2012),” *Journal of Interferon & Cytokine Research* 33 (2013): 49-51.

69 The National Archives (UK), DEFE 55/161:64-65.

70 The National Archives (UK), DEFE 55/377:51. The results of the vaccine field trials were published in Ch. Kunz, H.Hoffmann, and A. Stary, “Feldversuche mit einem Impfstoff gegen Frühsommer-Meningoencephalitis,” *Zentralblatt fur Bakteriologie, Parasitenkunde, Infektionskrankheiten und Hygiene. Erste Abteilung Originale. Reihe A: Medizinische Mikrobiologie und Parasitologie* 234 (1976): 141-144. The earlier report of Christian Kunz to the Austrian Ministry of Public Health and Environment in December 1974, however, mentions that initially the vaccine was only tested on 70 volunteers. See OeStA, 51.756/75.

71 Kunz, Hoffmann and Stary, “Feldversuche”; OeStA, 51.756/74.

72 OeStA, 51.176/74; Kunz, Hoffmann, and Stary, “Feldversuche,” 142.

73 OeStA, 51.176/74; 51.176/18-74.

74 My key sources here are declassified internal reports of the MRE Porton Down that, unfortunately, do not contain the names of specific scientists involved in the project.

75 The National Archives (UK), DEFE 55/211:28; Hammond and Carter, *From Biological Warfare,* 147-148.

76 The National Archives (UK), DEFE 55/225:22-23; OeStA, 51.176/74.

77 Radio interview of Christian Kunz, ORF Mittagsjournal, 7 April 1978, Österreichische Mediathek Audiovisual Archives, https://www.mediathek.at/katalogsuche/suche/detail/?pool=BWEB&uid=0725FAE6-3CD-000EC-00000D70-0725556A&vol=17544&cHash=1dfb7de834c6938cc5bf6c299cf55577.

78 The National Archives (UK), DEFE 55/225: 22-23; OeStA 51.176/78.

79 Christian Kunz, Franz X. Heinz, and Hanns Hofmann, “Immunogenicity and Reactogenicity of a Highly Purified Vaccine Against Tick-borne Encephalitis,” *Journal of Medical Virology* 6 (1980): 103–109.

80 The National Archives (UK), DEFE 55/225:22-23.

81 Immuno Aktiengesellschaft für Chemisch-Medizinische Produkte. Verfahren zur Herstellung von Frühsommer-Meningoenzephalitis-Virus (FSME-Virus) Vakzinen. AT Patent 358167, filed 22 December 1978, 9220/78, issued 25 August 1980.

82 Franz X. Heinz, Christian Kunz, and Hans Fauma, “Preparation of a Highly Purified Vaccine Against Tick-borne Encephalitis by Continuous Flow Zonal Ultracentrifugation,” *Journal of Medical Virology* 6 (1980): 213–221; Kunz, Heinz, and Hoffmann, “Immunogenicity and Reactogenicity.”

83 “Fünf Jahre Zeckenschutzimpfaktion,” *Österreichische Apotheker-Zeitung* 13 (1986): 266-271.

84 Hammond and Carter, *From Biological Warfare,* 148.

85 “Fünf Jahre Zeckenschutzimpfaktion,” 267; radio program “Zeckenschutzimpfung” in ORF Mittagsjournal 21 February 1994, Österreichische Mediathek Audiovisual Archives, https://www.mediathek.at/katalogsuche/suche/detail/?pool=BWEB&uid=114C9323-000-00120-00000BBC-114BC3E9&vol=89073&cHash=da8396dcf6463ae94503e4df5daecd6c.

86 Sarah Randolph, “Tick-Borne Encephalitis Incidence in Central and Eastern Europe: Consequences of Political Transition,” *Microbes and Infection* 10 (2008): 209–216; Dana Sumilo, Antra Bormane, Loreta Ašoklienė, Veera Vasilenko, “Socio-Economic Factors in the Differential Upsurge of Tick-borne Encephalitis in Central and Eastern Europe,” *Reviews in Medical Virology* 18 (2008): 81–95; Elinor Godfrey and Sarah Randolph, “Economic Downturn Results in Tick-borne Disease Upsurge,” *Parasites Vectors* 4: 35 (2011); A. Grzeszczuk, E. Sokolewicz-Bobrowska, D. Prokopowicz, “Adverse Reactions to Tick-Borne Encephalitis Vaccine: FSME-Immun,” *Infection* 26 (1998): 385-388; Andras Lakos et al., “Tick-borne Encephalitis,” *Parasitologia Hungarica* 29-30 (1996-1997): 5-16; D. Zavadska et al., “Tick-borne Encephalitis: A 43-year Summary of Epidemiological and Clinical Data from Latvia (1973 to 2016),” *PLOS ONE* 13: 11 (2018): e0204844.

